# Systematic Review and Meta-Analysis of Pancreatic Amylase Value on Postoperative Day 1 After Pancreatic Resection to Predict Postoperative Pancreatic Fistula

**DOI:** 10.1097/MD.0000000000002569

**Published:** 2016-02-08

**Authors:** Xiongxiong Lu, Xinjing Wang, Yuan Fang, Hao Chen, Chenghong Peng, Hongwei Li, Xiaxing Deng, Baiyong Shen

**Affiliations:** From the Pancreatic Disease Center; Research Institute of Pancreatic Disease; Shanghai Institute of Digestive Surgery, Ruijin Hospital, School of Medicine, Shanghai Jiao Tong University, Shanghai, China.

## Abstract

Early detection of postoperative pancreatic fistula (POPF) may help to improve the outcome following pancreatic surgery, and exclusion of POPF may allow early drain removal which can accelerate recovery. The aim of this study was to evaluate the diagnostic accuracy of drain/plasma pancreatic amylase values on postoperative day 1 (DPA1/PPA1) in POPF by means of a systemic review and meta-analysis.

Online journal databases and a manual search up to March 2015 were used. Studies clearly documenting DPA1 or PPA1 in predicting overall POPF (Grade 0 vs A+B+C) or clinically relevant POPF (Grade 0+A vs B+C) following pancreatic surgery were selected. Pooled predictive parameters were performed using STATA 12.0.

Fifteen studies were finally identified with a total of 4331 patients. The pooled sensitivity and specificity of DPA1 were 0.92 (95% confidence interval (CI) 0.81–0.96) and 0.77 (95% CI 0.64–0.86) for predicting overall POPF and 0.79 (95% CI 0.61–0.90) and 0.83 (95% CI 0.74–0.89) for predicting clinically relevant POPF. The pooled sensitivity and specificity of PPA1 were 0.74 (95% CI 0.63–0.82) and 0.62 (95% CI 0.55–0.70) for overall POPF. After the DPA1 at/over cutoff values for overall POPF or clinically relevant POPF, corresponding post-test probability (Post-test (+)) (if pretest probability was 50%) was 80% and 82% respectively, while, if values were below the cutoff values, the post-test probability (Post-test (−)) was 10% and 20% respectively. Post-test (+) and Post-test (−) of PPA1 for overall POPF were 66% and 30% respectively. In subgroup analysis, the summary sensitivities of cutoff <1000 group and cutoff >1000 group were 0.96 (0.92–0.98) and 0.85 (0.64–0.95), respectively; the summary specificities were 0.59 (0.44–0.72) and 0.86 (0.80–0.91) respectively. Positive LR were 2.3 (1.7–3.3) and 6.2 (3.7–10.2) respectively. Negative LR were 0.06 (0.03–0.14) and 0.18 (0.07–0.47) respectively.

DPA1 is a useful predictive test for overall POPF and clinically relevant POPF which has good sensitivity and specificity based on the current studies. Meanwhile, it should be cautiously applied to clinical practice because cutoffs had a wide range between studies.

## Abstract 2

Supplemental Digital Content is available in the text

## INTRODUCTION

Postoperative pancreatic fistula (POPF) remains the significant source and potentially life-threatening postoperative complication following pancreatic surgery which develops in 16% to 28% of patients.^1–7^ Given the frequency and severity of POPF, most surgeons today choose to place intraperitoneal drains with the aim of controlling anastomotic leakage.^8–12^ However, drain is a double-edged sword which may increase the risk of infection and the potential damage that may be induced by negative suction and erosion. To date, randomized controlled studies have provided compelling evidence that early drain removal (postoperative day (POD) 3 to 4) develops fewer complications when compared with late drain removal (POD >5).^13^ In clinical practice drains are normally removed at the surgeon's discretion when the risk of POPF has been excluded. The prediction of POPF can help to improve the management of abdominal drains, preventing an early or late removal.

Recently, in the field of pancreatic surgery, utilizing drain/plasma pancreatic amylase values on postoperative day 1 (DPA1/PPA1) as predictors of POPF to guide timing of drain removal catches high attention. DPA1 and PPA1 has been proposed with excellent sensitivity and specificity for overall POPF (Grade 0 vs A+B+C) and clinically relevant POPF (Grade 0+A vs B+C), but it was still controversial with some inconsistent views. This systematic review and meta-analysis aimed specifically to evaluate the value of DPA1 and PPA1 as predictors of POPF following pancreatic surgery.

## METHODS

Appropriate methods and standard guidelines for systematic reviews and meta-analyses of diagnostic test accuracy were followed.^14,15^ As the study is a meta-analysis, the ethical statement is not required.

### Study Selection

A computerized search was performed in MEDLINE (PubMed), Embase, the Cochrane Database, and the Cochrane Clinical Trials Registry to identify relevant articles published in the English language up to March 2015. The following search terms were used: “drain amylase,” “serum amylase,” “pancreatic fistula,” “early drain removal,” “pancreatic resection,” “sensitivity and pecificity.” Additional references were sought from the bibliographies of the selected articles and other recent reviews. Two researchers independently searched for articles. When discrepancies surfaced, a final consensus opinion was adopted after discussion or in consultation with a third investigator.

### Inclusion and Exclusion Criteria

Studies were included based on the following criteria: evaluation of the predictive value of DPA1 or PPA1 for POPF following pancreatic resection, and sufficient data to construct a 2×2 contingency table; an English language article published in a peer-reviewed journal. Abstracts, letters, editorials, expert opinions, reviews without original data and case reports and studies lacking sufficient data were excluded.

### Quality Assessment

The quality of included studies was assessed by using the Quality Assessment of Diagnostic Accuracy Studies (QUADAS) criteria which contains 14 questions.^16^

### Data Synthesis and Statistical Analysis

Measures of diagnostic accuracy, including cutoff values, sensitivity, and specificity of DPA1 or PPA1 for POPF and clinically relevant POPF, were recorded into a predesigned table. Meta-Disc 1.4 and STATA 12.0 were used for statistical analysis. From the 2 × 2 tables, summary sensitivity, specificity, positive likelihood ratio (LR), and negative LR (with corresponding 95% confidence interval) were calculated. Meanwhile, the area under the receiver operating characteristic curve (AUROC) was calculated to show the overall effectiveness of each test method. Pretest probabilities of 25%, 50%, and 75% versus corresponding post-test probabilities following a “positive” or “negative” PPA1 or DPA1 result were evaluated. The heterogeneity was evaluated by Cochran Q test and the inconsistency index (I^2^) which means the proportion of between-study difference besides chance variation. The random-effects model was used for meta-analysis and meta-regression was applied to find the potential heterogeneity sources if there was significant heterogeneity existing. Otherwise, the fixed effect model was applied. Meta-regression was performed using “metareg” in STATA. And publication bias was evaluated with Deeks funnel plot asymmetry test.

Two investigators (XW and XL) performed the data synthesis independently, and discrepancies were resolved by discussion or in consultation with a third investigator (XD).

## RESULTS

### Description of Studies

**FIGURE 1:**
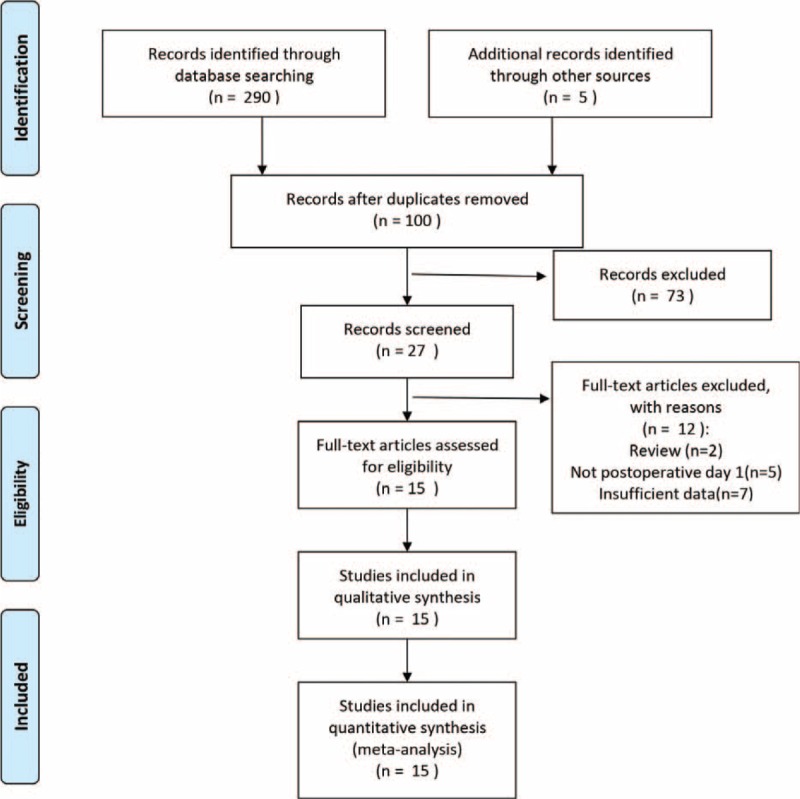
Flowchart showing selection of included studies for meta-analysis.

After the study search and selection, finally we identified 15 articles with a total of 4331 patients.^4,7,17–29^ The process of selecting trials for inclusion is shown in Figure 1. The characteristics of these 15 studies are shown in Table 1. Among all, there are 6 retrospective studies and 9 prospective studies. Twelve studies defined the POPF with the International Study Group on Pancreatic Fistula (ISGPF) standard and the other 3 studies used their own criteria (Table 1). Supplementary Figure 1 shows the methodological quality of included studies as evaluated by the QUADAS method.

**TABLE 1:**
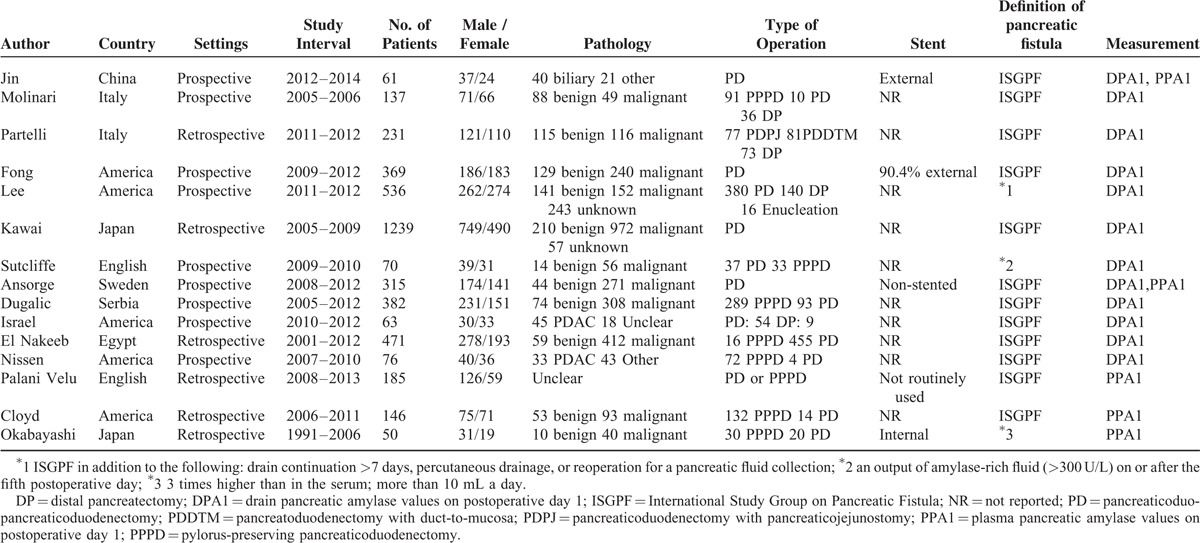
Characteristics of the Included Studies

Diagnostic indices of studies evaluating the DPA1 and PPA1 for pancreatic fistula are summarized in Tables 2 and 3, respectively. Twelve studies provided DPA1 results including 9 for overall POPF with the cutoffs ranging from 90 U/L to 5000 U/L and 4 for clinically relevant POPF with the cutoffs ranging from 1000 U/L to 4000 U/L. Five studies reported PPA1 results with 4 for overall POPF with the cutoffs ranging from 130 to 195 U/L and 2 for clinically relevant POPF with the 2 cutoffs (130 U/L, 177 U/L). Threshold effects of DPA1 for POPF were tested and showed there were no significantly threshold effects (Spearman correlation coefficient and *P* value of DPA1 for overall POPF were 0.65 and 0.06 respectively; Spearman correlation coefficient and *P* value of DPA1 for clinically relevant POPF were 0.40 and 0.60 respectively).

**TABLE 2:**
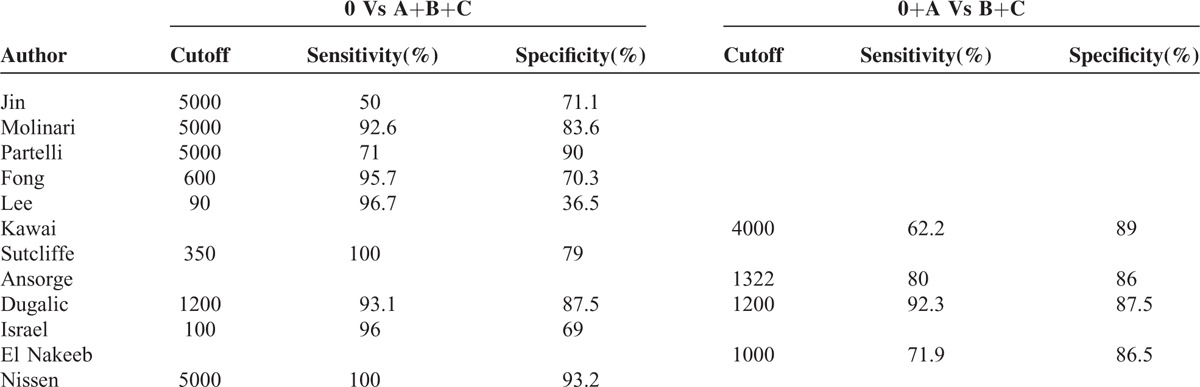
Diagnostic Indices of Studies Evaluating the DPA1 for Pancreatic Fistula

**TABLE 3:**
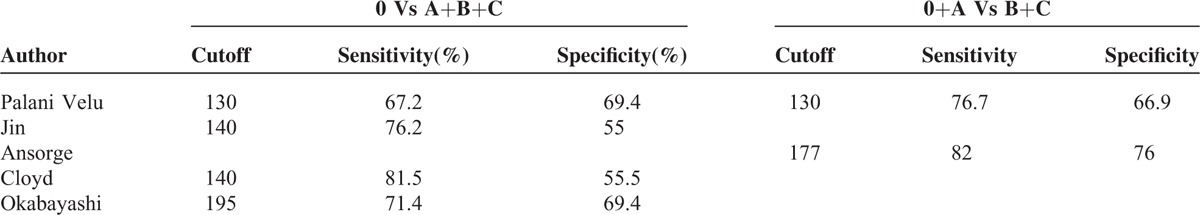
Diagnostic Indices of Studies Evaluating the PPA1 for Pancreatic Fistula

### Overall Diagnostic Indices

The pooled sensitivity and specificity of DPA1/PPA1 for POPF are shown in Table 4.

**TABLE 4:**
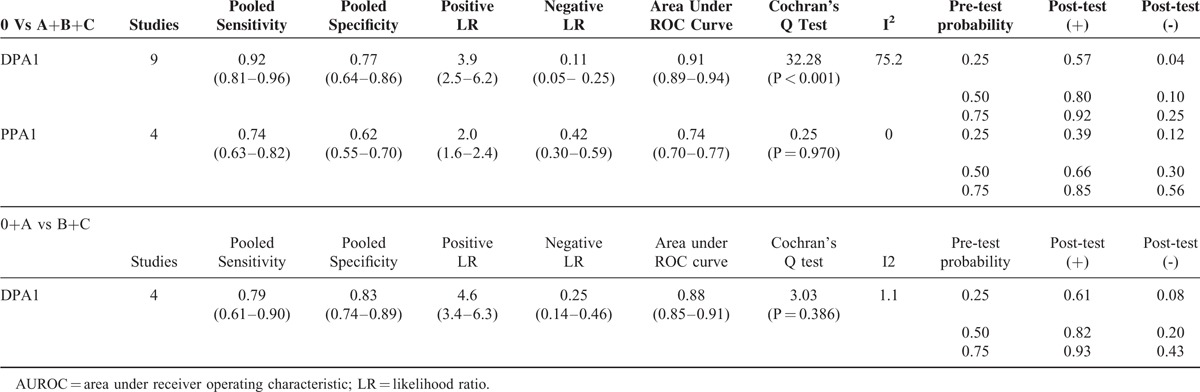
Meta-Analysis of Predictive Data for Overall Pancreatic Fistula (0 Vs A+B+C)/Clinically Relevant Pancreatic Fistula (0+A Vs B+C)

**FIGURE 2:**
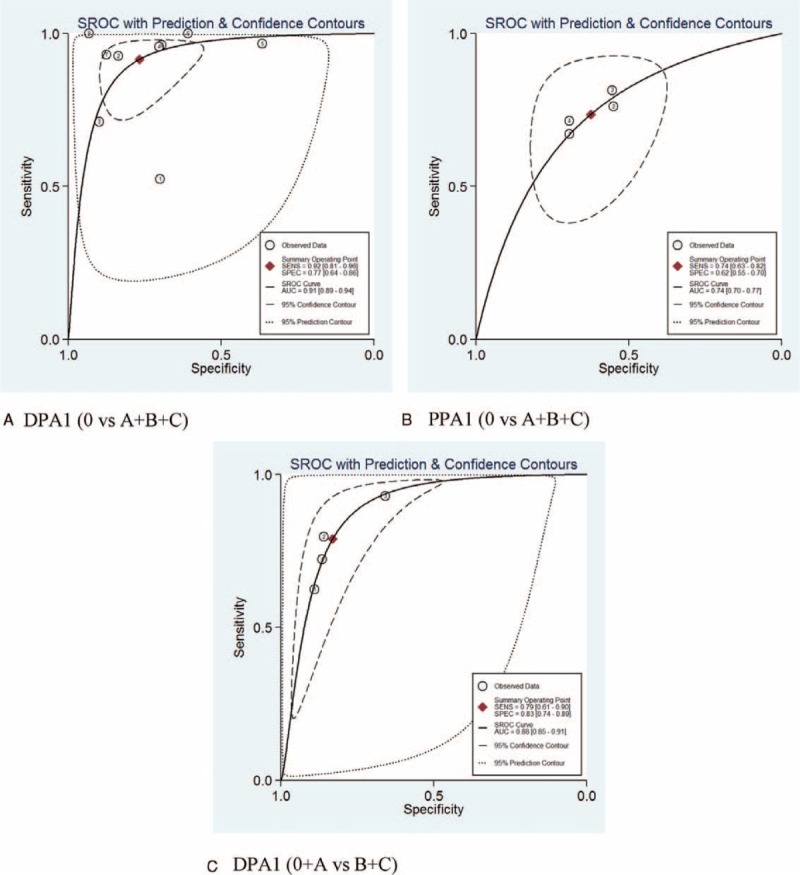
Receiver operating characteristic (ROC) curve analysis for the diagnosis of POPF (A, DPA1 for overall POPF. B, PPA1 for overall POPF. C, DPA1 for clinically relevant POPF). The hierarchical summary ROC (HSROC) curve and bivariable mean estimate (summary point) are shown, together with the corresponding 95% confidence region and 95% prediction region. The symbol size for each study is proportional to the study size.

For predicting overall POPF, the summary sensitivities of DPA1 and PPA1 were 0.92 (0.81–0.96) and 0.74 (0.63–0.82), respectively; the summary specificities were 0.77 (0.64–0.86) and 0.62 (0.55–0.70) respectively. Positive LR were 3.9 (2.5–6.2) and 2.0 (1.6–2.4) respectively. Negative LR were 0.11 (0.05–0.25) and 0.42 (0.30–0.59) respectively. AUROC were 0.91 (0.89–0.94) and 0.74 (0.70–0.77) respectively which is shown in Figure 2A and B. There was statistically significant heterogeneity of DAP1 (I^2^ = 75.2%, Cochran Q test = 32.28) which indicated significantly nonthreshold effects. To find the source of the heterogeneity, we applied meta-regression analysis to assess covariates from included studies. The “country,” “type of operation,” “stent,” and “definition of pancreatic fistula” were included. According to the meta-regression analysis, the main source of heterogeneity was country (*P* = 0.025).

For predicting clinically relevant POPF, pooled estimations of PPA1 could not be performed with only 2 studies that fit our criteria. Summary sensitivity and specificity of DPA1 for clinically relevant POPF were 0.79 (0.61–0.90) and 0.83 (0.74–0.89) respectively. Positive LR and negative LR were 4.6 (3.4–6.3) and 0.25 (0.14–0.46) respectively. AUROC was 0.88 (0.85–0.91) as in Figure 2C.

### Fagan Plot Analysis

The Fagan plot in DPA1 for overall POPF [17–22, 24–29] calculated that the positive post-test probabilities (post-test (+)) were 0.57, 0.80, 0.92 respectively, and the negative post-test probabilities (post-test (−)) were 0.04, 0.10, 0.25 respectively when the pretest probability was 25% or 50% or 75%. For PPA1 [22,27–29], the post-tests (+) were 0.39, 0.66, 0.85 respectively, and the post-tests (−) were 0.12, 0.30, 0.56 respectively.

For clinically relevant POPF, DPA1 [4,7,23,25] was demonstrated that the post-tests (+) were 0.61, 0.82, 0.93 respectively, and the post-tests (−) were 0.08, 0.20, 0.43 when the pretest probability was 25% or 50% or 75%.

**FIGURE 3:**
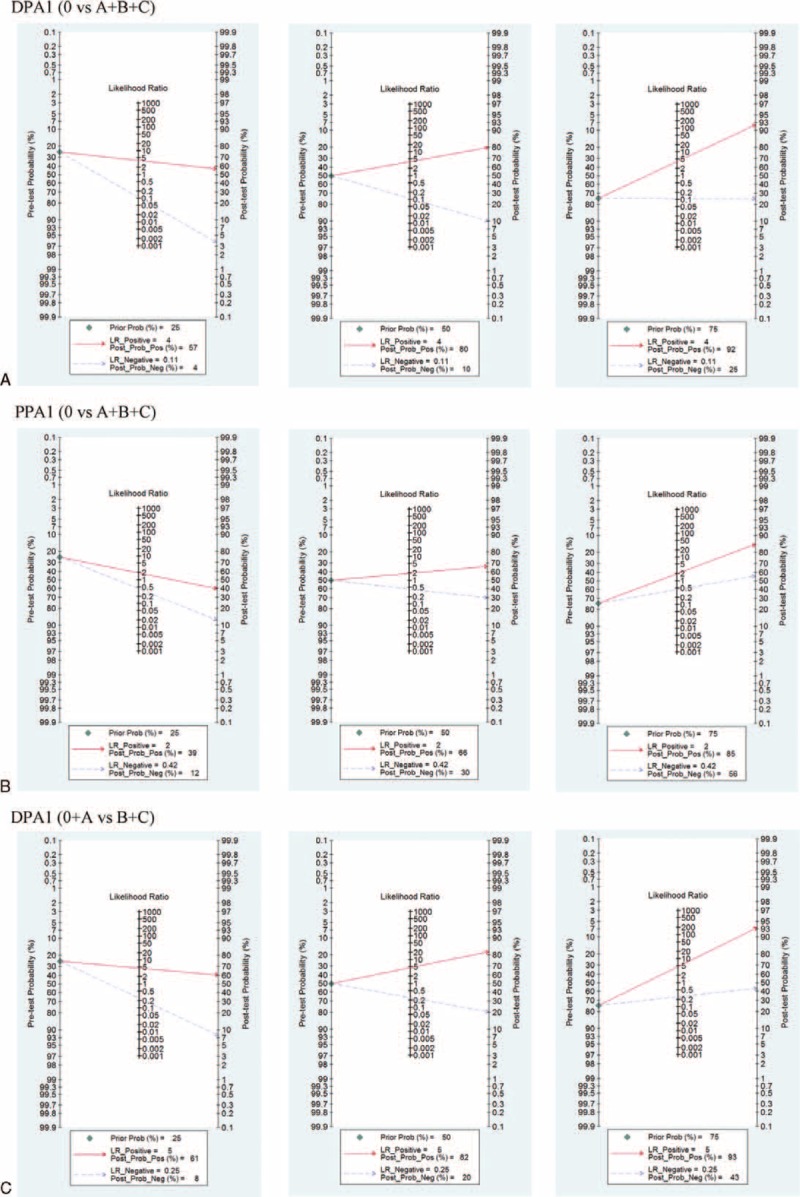
Fagan plot for the evaluation of clinical utilities (A, DPA1 for overall POPF. B, PPA1 for overall POPF. C, DPA1 for clinically relevant POPF).

Overall results of Fagan plot analysis are shown in Figure 3.

### Meta-Regression and Subgroup Analysis

For the wide range of cutoffs between studies when predicting overall POPF in DPA1, meta-regression analysis was performed to find the potential source. The “country,” “study interval,” “type of operation,” “stent,” and “definition of pancreatic fistula” were included. But finally there were no significantly heterogeneity sources (*P* values for country, study interval, type of operation, stent, and definition of pancreatic fistula were 0.076, 0.144, 0.384, 0.622 and 0.224, respectively).

For the various cutoffs among studies were really wide difference, subgroup analyses were performed. Overall diagnostic indices are shown in supplementary Table 1. The summary sensitivities of cutoff <1000 group and cutoff >1000 group were 0.96 (0.92–0.98) and 0.85 (0.64–0.95), respectively; the summary specificities were 0.59 (0.44–0.72) and 0.86 (0.80–0.91) respectively. Positive LR were 2.3 (1.7–3.3) and 6.2 (3.7–10.2) respectively. Negative LR were 0.06 (0.03–0.14) and 0.18 (0.07–0.47) respectively. AUROC were 0.96 (0.94–0.98) and 0.91 (0.88–0.93) respectively which was shown in supplementary Figure 2A and supplementary Figure 2B. Fagan plot analysis is shown in supplementary Figure 3. For overall POPF, the positive post-test probabilities (post-test (+)) were 0.44, 0.70, 0.88 respectively, and the negative post-test probabilities (post-test (−)) were 0.02, 0.06, 0.15 respectively when the pretest probability was 25% or 50% or 75% in cutoff <1000 group; the positive post-test probabilities (post-test (+)) were 0.67, 0.86, 0.95, respectively, and the negative post-test probabilities (post-test (−)) were 0.06, 0.15, 0.35, respectively when the pretest probability was 25% or 50% or 75% in cutoff >1000 group.

**FIGURE 4:**
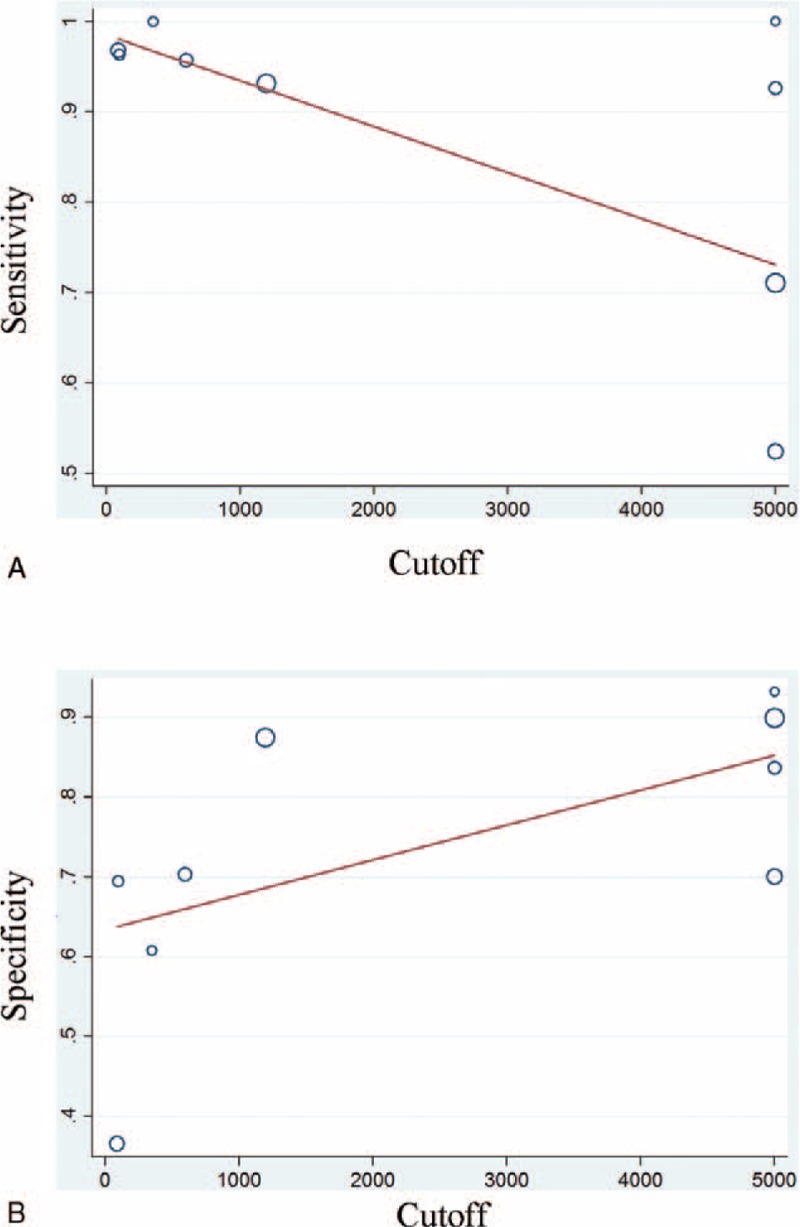
Meta regression of sensitivity (A) and specificity (B) on the cutoff in the studies (DPA1 for overall POPF). Open circles represent studies and sizes of the circles depend on the precision of each study estimate (ie, the inverse of its within-study variance). The line represents fitted values for the linear regression equation: sensitivity = −0.0000382 (SE 0.000) × cutoff + 0.982 (SE 0.070); specificity = 0.00047 (SE 0.000) × cutoff + 0.617 (SE 0.072).

Meta regression was performed using the cutoff in each study as an independent predictor of the estimated overall sensitivity, weighted inversely by the standard error of the sensitivity or specificity (Figure 4).

### Publication Bias

**FIGURE 5:**
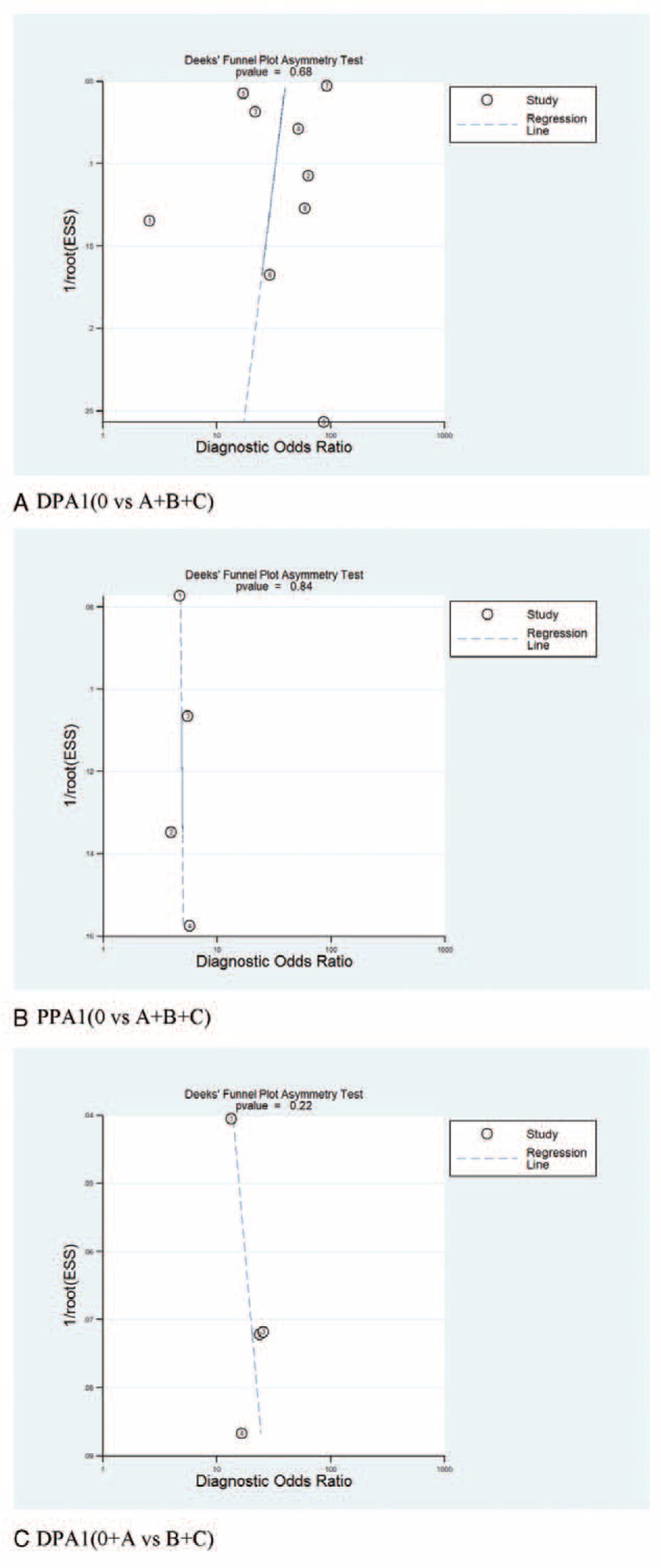
Deeks’ funnel plot asymmetry test for the evaluation of publication bias. (A, DPA1 for overall POPF. B, PPA1 for overall POPF. C, DPA1 for clinically relevant POPF.).

Deeks funnel plot asymmetry test of DPA1 and PPA1 for overall POPF and clinically relevant POPF is shown in Figure 5. There was no publication bias among the studies for all *P* values are more than 0.1.

## DISCUSSION

POPF is still the most relevant major complication which is associated with substantially increased life-threatening risks and financial resource utilization following pancreatic surgery. In this field, controversy about intraperitoneal drains has recently emerged. Recently, Kawai et al^30^ demonstrated that early removal of drains on POD4 had a significantly lower incidence of abdominal complications and POPF compared with those with drains still in place after POD8. Molinari et al^14^ proposed that DPA1 ≤5000 U/L identifies a subgroup of patients who may have a low possibility to develop the POPF and continued in place drain beyond the early postoperative period may be detrimental to them. Then in a randomized trial with 114 patients who underwent pancreatic resections, they demonstrated that in patients at low risk of POPF (DPA1 ≤ 5000 U/L) drains can be safely removed on POD 3 after surgery and a prolonged period of drains is associated with a higher incidence of complications.^13^

Successful management of a POPF often depends on its early prediction, there are few studies that have tried to evaluate the diagnostic value of DPA with the risk of developing POPF. Researchers have come up with different markers and models like DPA, PPA, CRP, WBC and many other indicators for the prediction.^1,5–7,17,31–33^ Molinari et al^21^ concluded that DPA1 > 5000 U/L was a significant predictive factor for POPF with a sensitivity and specificity of 93 and 84%, respectively. Sutcliffe et al^17^ reported that using 350 U/L as a cutoff, low DPA1 excluded POPF with sensitivity, specificity, positive predictive value, and negative predictive value of 100, 79, 41 and 100%, respectively. Ansorge et al^7^ proposed that combination of serum CRP and DPA adequately predicted the development of clinically relevant pancreatic fistula following pancreaticoduodenectomy (PD). For the comprehensive views, this present meta-analysis was designed to evaluate the diagnostic accuracy of DPA1/PPA1 in POPF based on the current published studies. As far as we know, this study is the first to evaluate the pooled performance of DAP1 and PPA1 for POPF.

In this meta-analysis of 15 studies, DPA1 showed the better discriminative capability than PPA1 in diagnosing both overall and clinically relevant POPF. And DPA1 not only had a high positive LR which means could be used as a rule-in diagnostic tool for the prediction of POPF, but also had a theoretically acceptable sensitivity and negative LR as a rule-out diagnostic tool. PPA1 had a relatively poor sensitivity and specificity when compared with DPA1, which may be caused by the limited number of included studies. Meanwhile, Fagan plot analysis was performed to evaluate the clinical utilities. Of PPA1 for overall POPF, when the pretest probability = 50%, there was only 66% probability of correctly diagnosing POPF with a positive result; however, the prediction would be wrong in 30% patients with a negative measurement. So, solo PPA1 was not an acceptable tool. DPA1 accurately diagnosed overall POPF in 80% patients with a positive measurement and misdiagnosis was present in only 10% with a negative result when the pretest probability = 50%. For clinically relevant POPF, 82% patients following positive results were correctly diagnosed by DPA1, while the diagnosis would be wrong in 20% patients with a negative measurement when the pretest probability = 50%. With regard to PPA1 for clinically relevant POPF, Fagan plot analysis was not performed due to insufficient studies (only 2 studies were included). Due to the current pooled results, DPA1 seems to be a theoretically acceptable diagnosis marker which is superior than PPA1. Of course, randomized studies should be performed to give more evidences and combined utilization of PPA1 and DPA1 was also welcomed to be applied.

Meta-analysis is not a widely identified method for pooling evidence from diagnostic studies; the authors believe that to some extent it provides valuable information for both clinicians and researchers until better studies are available. A major strength is that likelihood ratios and Fagan plot analysis have been reported in addition to pooled sensitivity, specificity, and AUROC values. Several limitations of this study should also be considered. First, the cut-off values had a wide range between studies which may induce a big bias. When talking about clinical practice, it is even more important to reach a consensus for which we need more prospectively designed studies to test. Second, there are few studies of high quality that provide unbiased data for our analysis. This is not only a limitation of our study, but the widespread application of prediction tools for POPF. It is urgent to further evaluate the value of DPA1 and PPA1 for POPF in a large, prospective, international, multicenter study. Another major limitation is the possibility of publication bias, in which surgeons who have had positive outcomes with diagnostic markers are more likely to publish their findings.

In conclusion, DPA1 is a useful predictive test for POPF and more high-quality studies should be carried to identify a clinically acceptable cutoff. Conversely, POPF can be excluded in patients who have a DPA1 less than cutoffs, and such patients may be candidates for early drain removal. In addition, more effective predictive markers and models are highly desirable to predict POPF.

## Supplementary Material

Supplemental Digital Content
