# Supplementary material for: Systematic Review and Meta-Analysis of Pancreatic Amylase Value on Postoperative Day 1 After Pancreatic Resection to Predict Postoperative Pancreatic Fistula
Source: Medicine (Baltimore). 2016 Feb 8;95(5):e2569. doi: 10.1097/MD.0000000000002569 (PMC4748879; doi:10.1097/MD.0000000000002569)

Supplementary Table 1 Meta-analysis of predictive data of DPA1 for overall pancreatic fistula (0 vs A+B+C)
LR, likelihood ratio; AUROC, area under receiver operating characteristic.

Cutoff	Studies	Pooled Sensitivity	Pooled Specificity	Positive LR	Negative LR	Area under ROC curve	Cochran's Q test	I^2	Pre-test probability	Post-test (+)	Post-test (-)
<1000 U/L	4	0.96 (0.92-0.98)	0.59 (0.44-0.72)	2.3 (1.7-3.3)	0.06 (0.03-0.14)	0.96 (0.94-0.98)	1.88 (*p*=0.598)	0	0.25	0.44	0.02
									0.50	0.70	0.06
									0.75	0.88	0.15
>1000 U/L	5	0.85 (0.64-0.95)	0.86 (0.80-0.91)	6.2 (3.7-10.2)	0.18 (0.07-0.47)	0.91 (0.88-0.93)	30.41(*p*<0.001)	86.8	0.25	0.67	0.06
									0.50	0.86	0.15
									0.75	0.95	0.35

Supplementary Fig. 1 The methodological quality of included studies as evaluated by the QUADAS method.

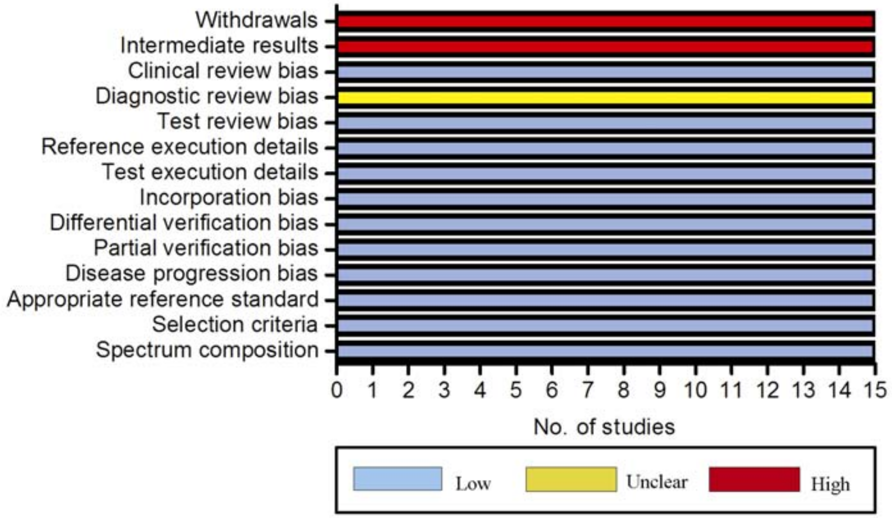

Supplementary Fig. 2 Receiver operating characteristic (ROC) curve analysis of DPA1 for the diagnosis of overall POPF (a. Cutoff < 1000U/L b. Cutoff > 1000U/L). The hierarchical summary ROC (HSROC) curve and bivariable mean estimate (summary point) are shown, together with the corresponding 95 percent confidence region and 95 percent prediction region. The symbol size for each study is proportional to the study size.

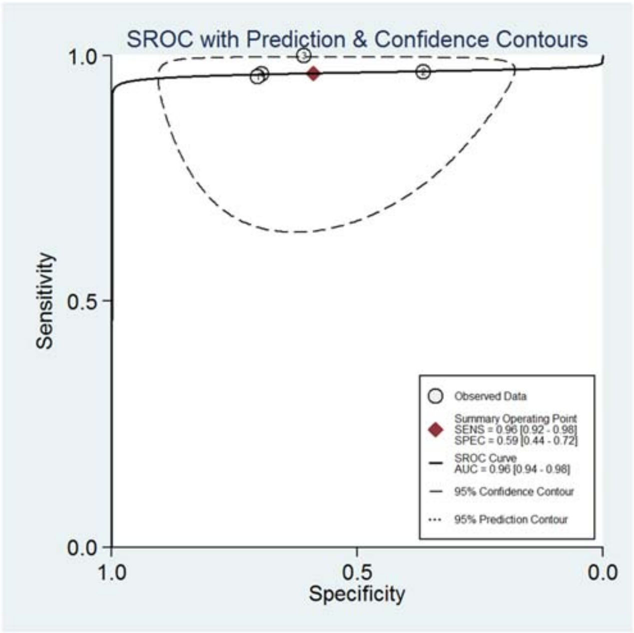

a. <1000 U/L

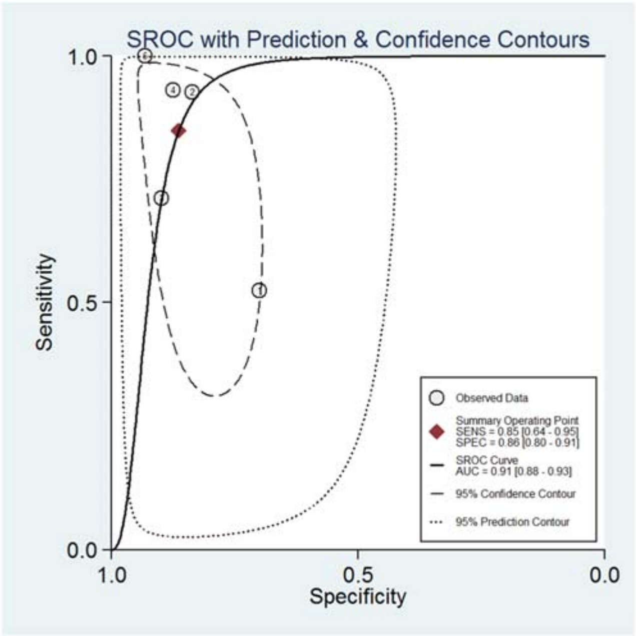

b. >1000 U/L

Supplementary Fig. 3 Fagan plot for the evaluation of clinical utilities (a. Cutoff < 1000U/L b. Cutoff > 1000U/L).

a. cutoff < 1000 U/L

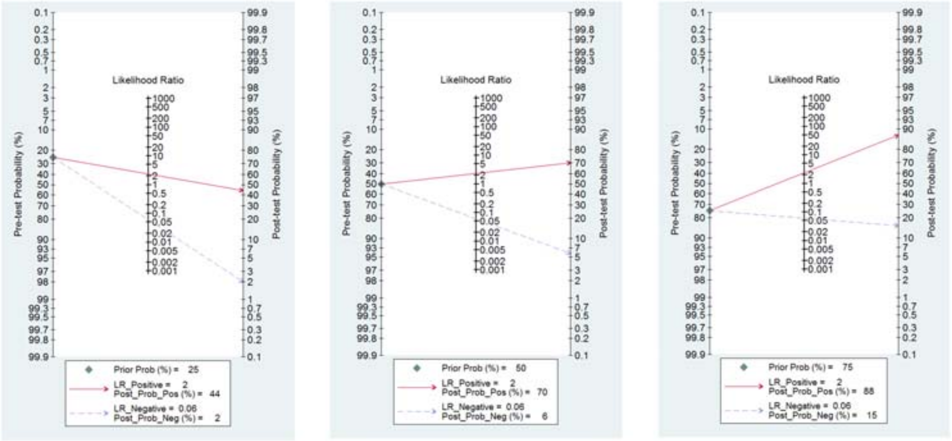

b. cutoff > 1000 U/L

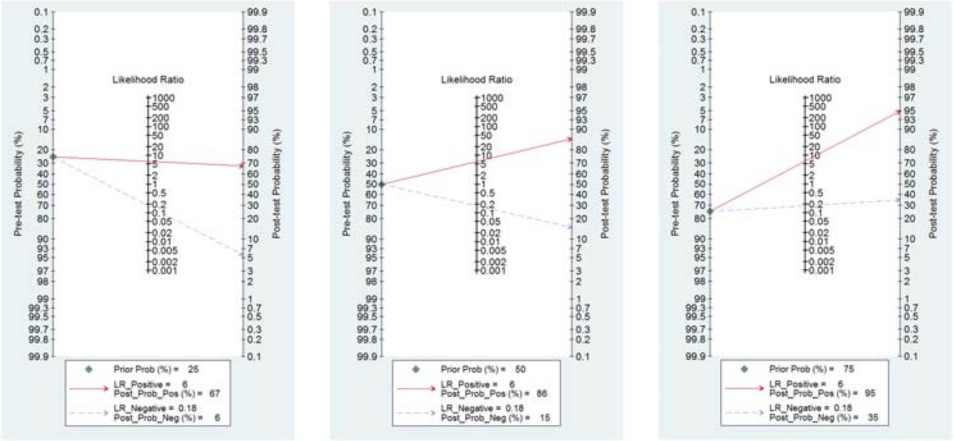

Supplement: Supplemental Digital Content [file medi-95-e2569-s001.pdf]
